# Fecal Immunochemical Test to Detect Colorectal Neoplasia in Lynch Syndrome: A Prospective Multicenter Study

**DOI:** 10.14309/ajg.0000000000003043

**Published:** 2024-08-20

**Authors:** Elsa L.S.A. van Liere, Nanne K.H. de Boer, Monique E. van Leerdam, Evelien Dekker, Maarten A.J.M. Jacobs, Jan Jacob Koornstra, Johan P. Kuijvenhoven, Margriet Lemmens, Gerrit A. Meijer, Manon C.W. Spaander, Beatriz Carvalho, Dewkoemar Ramsoekh

**Affiliations:** 1 Department of Gastroenterology and Hepatology, Amsterdam University Medical Center, Amsterdam, the Netherlands;; 2 Amsterdam Gastroenterology Endocrinology Metabolism Research Institute, Amsterdam, the Netherlands;; 3 Department of Gastrointestinal Oncology, Netherlands Cancer Institute, Amsterdam, the Netherlands;; 4 Department of Gastroenterology and Hepatology, Leiden University Medical Center, Leiden, the Netherlands;; 5 Cancer Center Amsterdam, Amsterdam, the Netherlands;; 6 Department of Gastroenterology and Hepatology, University Medical Centre Groningen, University of Groningen, Groningen, the Netherlands;; 7 Department of Gastroenterology and Hepatology, Spaarne Gasthuis, Hoofddorp, the Netherlands;; 8 Department of Pathology, Netherlands Cancer Institute, Amsterdam, the Netherlands;; 9 Department of Gastroenterology and Hepatology, Erasmus University Medical Center, Rotterdam, the Netherlands.

**Keywords:** Lynch syndrome, colorectal cancer, surveillance, biomarkers, fecal immunochemical test

## Abstract

**INTRODUCTION:**

Colonoscopy surveillance for Lynch syndrome is burdensome and postcolonoscopy colorectal cancers (CRCs) still occur. The noninvasive fecal immunochemical test (FIT) might guide optimal colonoscopy intervals.

**METHODS:**

Prospective, multicenter observational study in which individuals with Lynch syndrome performed a quantitative FIT before high-quality surveillance colonoscopy. Diagnostic performance of FIT at various thresholds ≤20 μg Hb/g feces was assessed for relevant neoplasia, including advanced neoplasia (CRC, advanced adenomas [AAs] and advanced serrated lesions [ASLs]) and non-advanced adenomas (NAAs).

**RESULTS:**

Of the 217 included individuals (59% female, median age 51 years), 4 had CRC, 5 AA, 4 ASL, and 57 NAA as most relevant neoplasia. The lowest FIT positivity threshold (2.5 μg Hb/g feces, 14% positivity rate) maximized detection: 4/4 CRCs, 4/5 AA, 1/4 ASL, and 9/57 NAA were detected, resulting in a sensitivity and negative predictive value of, respectively, 89% and 99% for CRC plus AA, 69% and 97% for advanced neoplasia, and 26% and 72% for all relevant neoplasia (91% specificity for all groups). At equal sensitivity and negative predictive value, specificity for advanced neoplasia optimized to 94% at threshold 4.1 μg/g. Per 100 FITs at threshold 4.1 μg/g, 11 individuals would test positive and thus proceed to colonoscopy, 2 individuals with advanced neoplasia would be missed and 3 individuals would need colonoscopy to detect 1 advanced neoplasia.

**DISCUSSION:**

FIT at thresholds ≤4.1 μg Hb/g feces may be a promising strategy to postpone colonoscopy in approximately 9 of 10 individuals with Lynch syndrome. Large validation studies that also provide gene variant-specific outcomes should be prioritized.

## Graphical abstract

**Figure:**
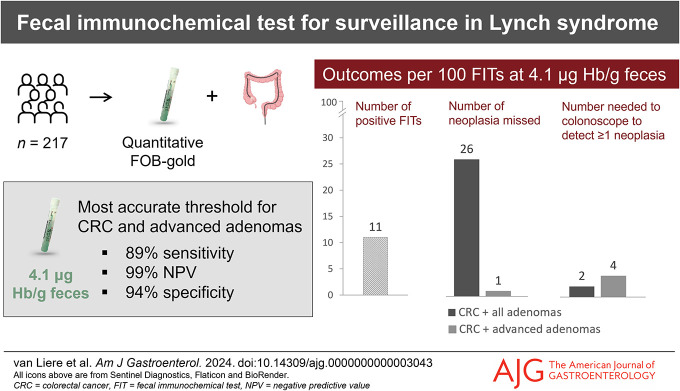

## INTRODUCTION

Affecting an estimated 1 in 279 individuals in the general population, Lynch syndrome is the most common inherited colorectal cancer (CRC) predisposition syndrome (1). Individuals with Lynch syndrome have an increased lifetime risk for CRC, ranging from 15% to 70% between the different germline mismatch repair gene variants (2). In this population, CRC incidence and mortality have reduced substantially through early detection of CRC and removal of precancerous adenomas, by means of biennial colonoscopy surveillance from age 25 years (2,3).

However, colonoscopy surveillance also poses several limitations. First, individuals with Lynch syndrome experience the regular and lifelong colonoscopies and required bowel preparation as time-consuming, burdensome, and detrimental to their quality of life (4,5). These barriers, among others, also lead to a concerning lack of adherence to timely surveillance; as many as 28% of individuals demonstrate delayed surveillance, which significantly undermines the effectiveness of the surveillance program (5–7). Another limitation is that colonoscopies are invasive, with a small risk for serious complications. Moreover, the procedures are costly and resource-intensive, therefore putting pressure on healthcare systems (8). All these limitations are further compounded by the fact that ∼70% of performed colonoscopies for Lynch syndrome do not reveal neoplasia requiring removal (9,10), thus were “unnecessary” in retrospect. Hence, it would be valuable to reduce the number of colonoscopies in those individuals unlikely to benefit from colonoscopy. On the other hand, some individuals may benefit from a shorter colonoscopy interval because postcolonoscopy CRCs still occur (11).

In this context, noninvasive biomarkers might predict the presence of colorectal neoplasia and therefore select individuals for colonoscopy. A potential diagnostic biomarker test for CRC and advanced adenomas in Lynch syndrome is the fecal immunochemical test (FIT), a high-throughput, low-cost, and patient-friendly test which has been extensively studied in other populations and implemented in many CRC screening programs worldwide (12,13). In individuals at increased risk of CRC, FIT has shown high sensitivity for CRC—particularly with thresholds ≤20 μg Hb/g feces—as well as for advanced adenomas on thresholds ≤4 μg Hb/g feces (14–16). Despite the routine use of FIT in other populations, the role of FIT for Lynch syndrome has not yet been determined.

Therefore, we designed a prospective multicenter observational study to evaluate diagnostic performance of FIT for CRC and precancerous lesions at different positivity thresholds in Lynch syndrome.

## METHODS

### Study design

This prospective multicenter observational study was performed from February 2021 to February 2023 at 5 hospitals across the Netherlands—3 academic hospitals (Amsterdam University Medical Center, Erasmus University Medical Center, University Medical Center Groningen), 1 comprehensive cancer center (Netherlands Cancer Institute), and 1 general hospital (Spaarne Gasthuis). The study was approved by the Research Ethical Committee of Amsterdam UMC (2020.317), and by local ethical committees of the other participating centers, and was registered at the WHO International Clinical Trials Registry Platform (NL8749). Written informed consent was obtained from all study participants.

### Study participants and sample collection

We invited consecutive individuals with a pathogenic germline variant in one of the mismatch repair genes or *EPCAM*, who were scheduled for surveillance colonoscopy. In the Netherlands, colonoscopy surveillance for Lynch syndrome starts at age 25 years (independent of gene variant) and is generally performed every 2 years, with some exceptions at 1-year intervals. The Dutch guideline for Lynch syndrome does not recommend chemoprevention by means of aspirin (an agent that could have skewed FIT results). Individuals who had a history of bowel resection could be included in our study, except for (sub)total colectomy.

Individuals participating in our study were asked to perform a FIT in the 3 months before surveillance colonoscopy. FIT was performed before bowel preparation using the “FecesCatcher” (TAG Hemi, Zeijen, the Netherlands), which eases sample collection and prevents contamination from urine and toilet water. FITs were collected alongside fecal samples for another study, and therefore stored in participant's own freezer, within 1 hour after stool collection. Subsequently, samples were transported to the hospital either by the participant using cooling gel packs (200 × 282 mm segmented; De Ridder Packaging, Assendelft, the Netherlands) or by a researcher using dry ice. On arrival at the hospital, FITs were stored at −20 °C until further analysis. Although freezing FIT is not a standard approach in other studies and CRC screening programs, we previously observed that hemoglobin levels measured by FIT were not affected by freeze-thaw cycles (17).

Individuals were excluded if during surveillance colonoscopy the cecum landmarks or ileocolic anastomosis were not identified, the Boston Bowel Preparation Score was not ≥2 in each segment, the endoscopist observed inflammation or infection (e.g., diverticulitis or active inflammatory bowel disease), or polypectomy was performed, but no histopathology report was available. Other exclusion criteria were bowel preparation within 7 days before sample collection or insufficient fecal sample for analysis.

### Colonoscopy and histopathology

Colonoscopies were either performed or supervised by consultant gastroenterologists, who had performed more than 2,000 colonoscopies. In line with the European Society of Gastrointestinal Endoscopy guideline for endoscopic management of Lynch syndrome, colonoscopies were performed using high-definition white light endoscopy, with additional use of advanced imaging techniques at the discretion of the endoscopist (2). Following the European Society of Gastrointestinal Endoscopy quality measures, minimum withdrawal time was 6 minutes (18). Except from obvious hyperplastic lesions ≤5 mm in the rectosigmoid (19), all detected neoplasia were resected using standard polypectomy techniques. Histopathology was evaluated by experienced gastrointestinal pathologists and was reported according to the Vienna classification of gastrointestinal neoplasia (20). Serrated lesions were classified according to the 2019 World Health Organization criteria as sessile serrated lesion (SSL, with or without dysplasia), traditional serrated adenoma, or hyperplastic polyp (21).

### FIT analysis

Similar to the Dutch population-based CRC screening program, we used the SENTiFIT—fecal occult blood (FOB) gold test (Sentinel Diagnostics, Milan, Italy) for quantitative fecal hemoglobin analysis. FITs were analyzed in 4 batches after a median storage of 340 days (interquartile range [IQR] 169–432) and with no difference in positivity rates between batches (*P* = 0.691), using an automatic analyzer (SENTiFIT 270; Sentinel Diagnostics) according to manufacturer's protocols. The analyzer was calibrated, and checked for quality control at the beginning of each batch analysis, and had a lower limit of quantitation of 2.5 μg Hb/g feces and a lower limit of detection of 1.8 μg Hb/g feces. Laboratory personnel was blinded with respect to colonoscopy results.

### Definitions and outcomes

Individuals were classified by their most relevant neoplasia at colonoscopy. Relevant neoplasia included CRC, advanced adenoma (defined as adenomas ≥10 mm, with (tubulo)villous histology or showing high-grade dysplasia (22)), advanced serrated lesion (defined as serrated lesions ≥10 mm or showing dysplasia (23)), or non-advanced adenoma. Individuals with non-advanced serrated lesions only, along with those having no colorectal lesions, were deemed as controls, because recent research showed that they have a negligible risk for metachronous CRC within the 3 years after colonoscopy (24). Neoplasia size, location, and the Paris classification for morphology were retrieved from the endoscopy report; neoplasia located in the cecum, ascending colon and transverse colon, were classified as proximal, whereas neoplasia in the splenic flexure, descending colon, sigmoid, and rectum as distal.

Diagnostic performance of FIT was evaluated at different positivity thresholds—ranging between the lower limit of quantitation and the 20 μg Hb/g feces threshold which is used in most CRC screening programs worldwide (12,13)—and for different outcome measures: all relevant neoplasia (CRC, advanced serrated lesions, all adenomas), advanced neoplasia (CRC, advanced adenomas, advanced serrated lesions), and CRC plus advanced adenomas.

### Statistical analysis

Data were presented as medians with IQRs or as numbers with percentages. Differences in baseline demographics between the neoplasia and control group were assessed using the Mann–Whitney *U* test for continuous variables and the χ^2^ or Fisher exact test (in case ≥20% of cells had expected counts less than 5) for categorical variables.

Using IBM SPSS Statistics version 28, diagnostic performance of FIT was calculated in terms of sensitivity, specificity, negative predictive value (NPV), positive predictive value (PPV), and the area under the curve including the corresponding *P* value. Receiver operating characteristic analysis established the most accurate threshold. Fagan's nomogram in RStudio version 4.2.1 visualized the estimated probability that an individual has neoplasia following negative or positive FIT, with the neoplasia prevalence being considered as the pretest probability (25). Finally, per 100 individuals tested by FIT at various thresholds, we calculated the number of missed neoplasia and the number needed to colonoscope—that is, the number of individuals undergoing colonoscopy to detect at least 1 neoplasia, a simple indicator of cost-effectiveness. Calculations were based on the neoplasia prevalence as observed in our cohort, given that no other data are available using our definition of relevant neoplasia in Lynch syndrome.

### Patient and public involvement

The design and conduct of the study were established in collaboration with the Dutch Lynch Polyposis patient association. Patient and public representatives of the Dutch Digestive Foundation provided feedback during the grant application. The results of this study will be shared to the public through scientific conferences, press releases, and the Dutch Lynch Polyposis patient association. The results will also be disseminated to individuals with Lynch syndrome that participated in our study.

## RESULTS

### Patient characteristics

**Figure 1.**
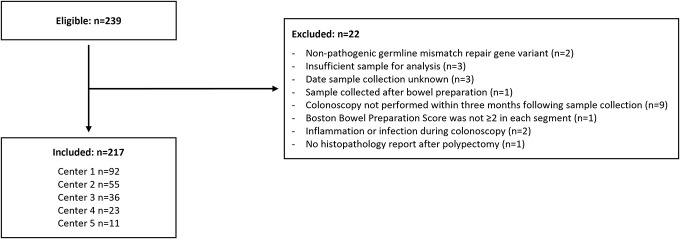
Flowchart showing the selection of the study population.

In total, 217 individuals were included in the study (Figure 1, Table 1). Of the inclusions, 128 (59%) were female, median age was 51 years (IQR 41–61), and 33 (15%) had a personal history of CRC. Pathogenic germline variants in *MSH6* were the most common, followed by *PMS2* and *MSH2* variants, and then *MLH1* and *EPCAM* variants. Most individuals (79%) had undergone at least 2 colonoscopies before inclusion, with a median time since last colonoscopy of 24 months (IQR 23–27). FITs were performed a median of 7 days (IQR 4–14) before colonoscopy.

**Table 1.**
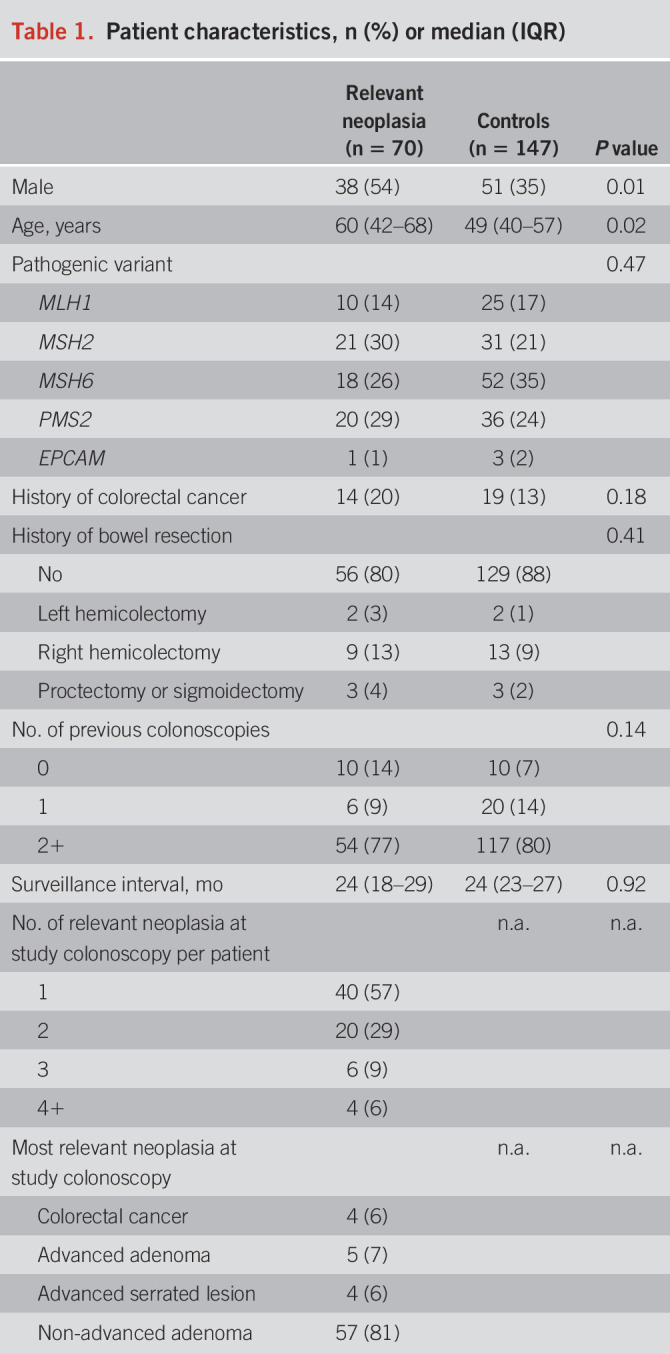
Patient characteristics, n (%) or median (IQR)

### Neoplasia characteristics

Of the 217 individuals, 70 (32%) had relevant neoplasia at colonoscopy; the vast majority had no more than 2 relevant neoplasias and the most advanced relevant neoplasia was CRC in 4/217 (1.8%; all adenocarcinomas), advanced adenoma in 5/217 (2.3%; all ≥10 mm with low-grade dysplasia), advanced serrated lesion in 4/217 (1.8%; 1 small SSL with dysplasia and 3 SSLs ≥10 mm without dysplasia), and non-advanced adenoma in 57/217 (26.3%). Of the 4 CRCs, 2 were stage I (1 distal pT1N0 and 1 proximal pT2N0) and 2 were stage III (1 proximal cT2N1 and 1 proximal cT3N1). The advanced serrated lesions and adenomas were located in the proximal colon in 55% and had mostly sessile (56%) or flat (26%) morphology; for further characteristics, see Supplementary Table S1 (Supplementary Digital Content 1, http://links.lww.com/AJG/D384). Compared with controls, individuals having relevant neoplasia were older and more frequently of male sex (*P* value 0.02 and 0.01, respectively, Table 1); no other significant differences in baseline characteristics were found.

The 13 advanced neoplasia were identified in 5/52 (9.6%) *MSH2* carriers, 2/35 (5.7%) *MLH1* carriers, 3/56 (5.4%) *PMS2* carriers, and 3/70 (4.3%) *MSH6* carriers; all 4 advanced serrated lesions were found in *PMS2* and *MSH6* carriers. Of these 13 individuals, 8 (62%) were male, median age was 61 years (IQR 42–67), and 3 (23%) had a personal history of CRC and subsequent right hemicolectomy. Regarding surveillance history, 4 individuals (31%) had not undergone any previous colonoscopy whereas the other 9 individuals had, with a median time since last colonoscopy of 28 months (IQR 21–60). Of the 4 CRCs, 2 were found during the index colonoscopy whereas the other 2 were postcolonoscopy CRCs after a delayed colonoscopy surveillance interval of 61 and 73 months.

### Fecal hemoglobin concentration

Fecal hemoglobin was detectable in 4/4 CRCs, 4/5 advanced adenomas, 1/4 advanced serrated lesions, and 9/57 nonadvanced adenomas. In these individuals, median hemoglobin concentration per gram feces was 240.3 μg (range 6.3–1,361.3) for those with CRC, 18.6 μg (range 6.2–87.0) for those with an advanced adenoma, 4.4 μg for the patient with an advanced serrated lesion, and 5.9 μg (range 2.8–10.2) for those with a non-advanced adenoma. Owing to the low number of individuals in each neoplasia group, we were not able to investigate whether hemoglobin concentration varied statistically between groups.

### Diagnostic accuracy: all relevant neoplasia

**Figure 2.**
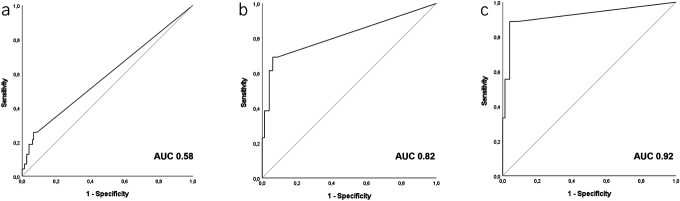
Receiver operating characteristic curves showing the diagnostic accuracy of the fecal immunochemical test for (**a**) all relevant neoplasia; CRC, advanced serrated lesions, all adenomas, (**b**) advanced neoplasia; CRC, advanced serrated lesions, advanced adenomas; and (**c**) CRC plus advanced adenomas. AUC, area under the curve; CRC, colorectal cancer.

Using the lowest positivity threshold of 2.5 μg Hb/g feces, FIT identified 4/4 CRCs, 4/5 advanced adenomas, 1/4 advanced serrated lesions, and 9/57 non-advanced adenomas, resulting in a 26% sensitivity and 72% NPV (Table 2, Figure 2). Of note, 6/24 (25%) distally located and 3/33 (9%) proximally located non-advanced adenomas were identified, respectively (*P* = 0.15). The accuracy to detect any type of neoplasia did not improve on other thresholds ≤20 μg Hb/g feces, with the 20 μg/g threshold specifically identifying only 3/4 CRCs, 2/5 advanced adenomas, 0/4 advanced serrated lesions, and 0/57 non-advanced adenomas, corresponding to a 7% sensitivity and 69% NPV. For all thresholds, the estimated probability of having relevant neoplasia after negative FIT was more or less comparable to the pretest probability of 32%, in other words, a negative FIT does not change the probability that an individual has relevant neoplasia (see Supplementary Figure S1, Supplementary Digital Content 1, http://links.lww.com/AJG/D384).

**Table 2.**
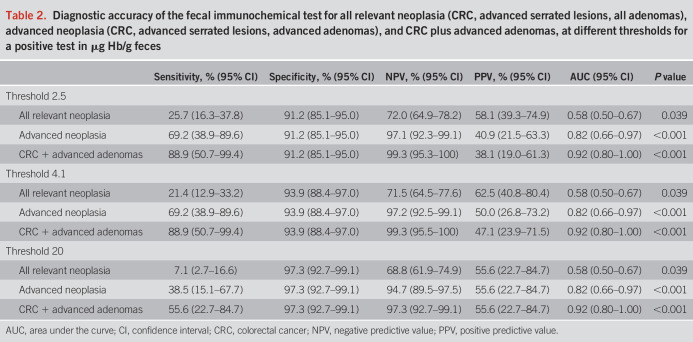
Diagnostic accuracy of the fecal immunochemical test for all relevant neoplasia (CRC, advanced serrated lesions, all adenomas), advanced neoplasia (CRC, advanced serrated lesions, advanced adenomas), and CRC plus advanced adenomas, at different thresholds for a positive test in μg Hb/g feces

### Diagnostic accuracy: advanced neoplasia

When advanced neoplasia were the target lesions, the 2.5 μg Hb/g feces threshold demonstrated 69% sensitivity, 91% specificity, 97% NPV, and 41% PPV. The most accurate threshold was found to be 4.1 μg Hb/g feces: specificity and PPV improved to 94% and 50%, respectively, while not compromising sensitivity and NPV (Table 2, Supplementary Figure S2, see Supplementary Digital Content 1, http://links.lww.com/AJG/D384). Again, the 20 μg Hb/g feces threshold showed poor diagnostic accuracy as sensitivity was only 39% (NPV was 95%).

### Diagnostic accuracy: CRC and advanced adenomas

**Figure 3.**
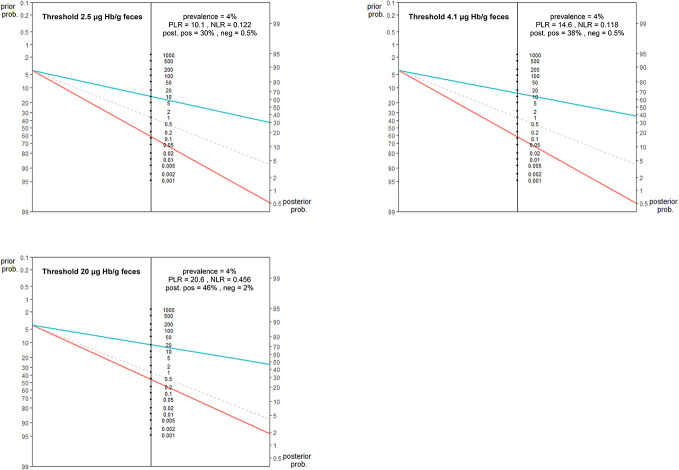
Fagan's nomograms illustrating the pretest and post-test probability of colorectal cancer (CRC) and advanced adenomas (AA), following positive (blue line) and negative (red line) fecal immunochemical test (FIT) at different thresholds. The gray line represents the situation when the post-test probability remains unchanged. The figures show that a negative FIT at thresholds ≤ 4.1 μg Hb/g feces decreases the estimated probability of CRC and AA from 4% to 0.5%, while a positive FIT increases the probability of these neoplasia to 30% (2.5 μg/g threshold) or 38% (4.1 μg/g threshold). By contrast, a negative FIT at 20 μg Hb/g feces decreases the probability of CRC and AA only to 2%. NLR, negative likelihood ratio; PLR, positive likelihood ratio.

When CRC and advanced adenomas were the target lesions, the 2.5 μg/g threshold showed 89% sensitivity, 91% specificity, 99% NPV, and 38% PPV. Again, specificity and PPV improved at the 4.1 μg/g threshold—to 94% and 47%, respectively—while not compromising sensitivity and NPV (Table 2, Figure 3). By contrast, FIT at 20 μg/g showed a sensitivity of only 56% with a 97% NPV.

### Outcomes per 100 individuals tested by FIT

**Figure 4.**
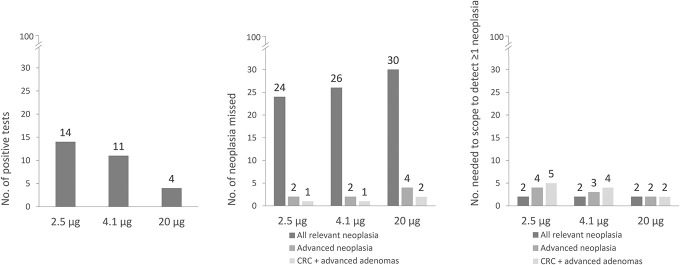
Number of positive tests, lesions missed and needed to scope to detect at least 1 neoplasia per 100 individuals tested by fecal immunochemical test at different thresholds in μg Hb/g feces. Calculations are based on a prevalence of 32% for all relevant neoplasia (CRC, advanced serrated lesions, all adenomas), 6% for advanced neoplasia (CRC, advanced serrated lesions, advanced adenomas), and 4% for CRC plus advanced adenomas. CRC, colorectal cancer.

Per 100 individuals with Lynch syndrome tested by FIT, a positive test result would be obtained in 14 individuals (2.5 μg/g threshold), 11 individuals (4.1 μg/g threshold), and 4 individuals (20 μg/g threshold, Figure 4). The number of missed relevant neoplasia would vary between 24 and 30 lesions depending on the threshold used, while for all 3 thresholds the number of individuals undergoing colonoscopy to detect at least 1 relevant neoplastic lesion would be 2. On the other hand, per 100 FITs, only 2 individuals with an advanced adenoma or advanced serrated lesion would be missed using either the 2.5 or 4.1 μg/g threshold, with a number needed to scope of 4 individuals or 3 individuals for the 2.5 or 4.1 μg/g threshold, respectively.

## DISCUSSION

This is the first prospective multicenter study evaluating the performance of FIT in Lynch syndrome, a population requiring strict lifelong surveillance that would benefit substantially from a noninvasive, low-cost biomarker guiding optimal surveillance colonoscopy intervals. Our study shows that FIT at threshold 4.1 μg Hb/g feces maximized detection of CRC and advanced adenomas to 89% sensitivity and 99% NPV, along with 94% specificity. Per 100 individuals tested by FIT at 4.1 μg/g, 11 individuals would proceed to colonoscopy while only 1 individual with advanced adenoma would be missed, with a high efficiency in terms of number needed to colonoscope (n = 4) as surrogate measure for cost-effectiveness. The 20 μg Hb/g feces threshold, which is used in most CRC screening programs, detected CRC and advanced adenomas with only 56% sensitivity.

In population-based CRC screening, the goal of FIT is to accurately detect CRC, and therefore, a high test specificity for CRC is prioritized over adenoma detection. However, in Lynch syndrome, FIT should detect CRC as well as advanced adenomas and, ideally, non-advanced adenomas with high sensitivity and NPV, accepting a suboptimal specificity. Our study confirms previous research showing that accuracy of FIT to detect CRC and advanced adenomas is highly dependent on the FIT threshold, with sensitivity improving significantly—although at the expense of specificity—the lower the threshold, predominantly through enhanced detection of adenomas (14–16,26). In line with our study, thresholds ≤20 μg Hb/g feces have shown high sensitivity and specificity for CRC in recent meta-analyses which evaluated either average-risk individuals or individuals with a familial or personal history of CRC (15,26). On the other hand, a threshold as low as the limit of detection (2 μg Hb/g feces) seemed to be required to detect advanced adenomas with high sensitivity in individuals under colonoscopy surveillance (14,16). A slightly higher threshold of 6 μg Hb/g feces resulted in suboptimal sensitivity when individuals with Lynch syndrome were tested by FIT as an emergency service during the coronavirus pandemic, although accuracy was based on only 123 individuals without reporting detailed patient characteristics and the number of CRCs and advanced adenomas (27).

Regardless of the FIT threshold, we found that FIT has poor diagnostic accuracy for both non-advanced adenomas and advanced serrated lesions, which seemed to be more pronounced for lesions located in the proximal colon. These findings are in line with those in previous studies and may be explained by the low tendency of such polyps to bleed (14,28–30). While identification of non-advanced adenomas is relevant in Lynch syndrome given the accelerated progression of adenomas to CRC (31), the independent risk of serrated lesions in this population remains unknown. Nevertheless, the prevalence of serrated lesions in individuals with Lynch syndrome seems to be similar to that of the general population (32), in whom most advanced serrated lesions have no dysplasia (24,28) and probably little to no malignant potential in the short term (24,33–35), granting a window for their detection. To improve sensitivity of FIT for advanced serrated lesions and non-advanced adenomas in Lynch syndrome, potential strategies might be to sample 2 FITs per patient, repeat FIT at short intervals, or add other biomarkers such as stool DNA (16,36–39). When devising novel biomarkers, the molecular pathways of colorectal tumorigenesis identified in Lynch syndrome should be considered (40).

Even if sensitivity for advanced serrated lesions and non-advanced adenomas remains poor, FIT might still enable personalized surveillance in Lynch syndrome by selecting those high-risk or low-risk carriers who might benefit from either a shorter or longer colonoscopy interval, respectively. Although risk group-specific performance of FIT could not be assessed in our study, individuals having variants in the higher-risk genes *MLH1* or *MSH2* (11) could potentially be tested with FIT halfway through the 2-yearly colonoscopy interval, aiming to reduce the postcolonoscopy CRC rate. *MLH1* and *MSH2* variants accounted for all CRCs and most advanced adenomas in our study, although these variants were under-represented because of their lower prevalence (1) and (sub)total colectomy being an exclusion criterion. For individuals at lower risk for neoplasia—such as those having *MSH6* or *PMS2* variants (1,11,41–43) and no other risk factors for CRC (e.g., personal history of colorectal neoplasia, smoking, and overweight) (6,44,45)—a negative FIT could potentially extend the 2-year surveillance interval with, for example, 6–12 months, or even further if subsequent FITs also yield negative results. Reducing colonoscopy frequency could be of great benefit for lower-risk individuals, provided that CRC prevention remains intact through accurate detection of advanced adenomas.

Ideally, future clinical studies should compare the effectiveness and harm of fixed colonoscopy intervals with FIT-guided colonoscopy intervals. Such studies should evaluate both the FOB-gold test and the other widely used quantitative FIT called the OC sensor (which has a slightly higher limit of quantitation of 4–5 μg Hb/g feces), as well as high-quality qualitative FITs at specific very low thresholds as they become available (46,47). Various thresholds that have precision to 0.1 μg should be assessed to determine the optimal threshold, which should be an integer to be practical in clinical practice. Regarding outcome measures, such studies should evaluate neoplasia detection rates and (modelled) long-term impact on CRC incidence and mortality per risk group, as well as patient acceptability and adherence over multiple surveillance rounds. Although a recent study showed that the uptake of 3 rounds of FIT was similar to 1-time colonoscopy in surveillance-naïve individuals with familial CRC (48), uptake of FIT may be superior when taking multiple surveillance rounds into account, especially in individuals with Lynch syndrome who require surveillance more frequently than those with familial CRC. Moreover, individuals with Lynch syndrome have shown to be receptive to noninvasive, less-burdensome surveillance strategies (5). Other aspects that a clinical trial should compare are complication rates, costs, and burden to health systems including endoscopy practice. That endoscopy capacity can be a bottleneck became unmistakably evident during the coronavirus pandemic, although FIT demonstrated clinical value in prioritizing individuals with Lynch syndrome for colonoscopy (27,49).

This study has several strengths, as well as some limitations. A strong aspect is that it is the first prospective multicenter study assessing the diagnostic performance of FIT for Lynch syndrome, with evaluation of different positivity thresholds in a cohort of 217 individuals. Other strengths are the consecutive recruitment of participants and that all participants underwent high-quality colonoscopy as the reference standard (50), thereby limiting the possibility of spectrum bias. Moreover, neoplasia prevalence was comparable with other high-quality studies in Lynch syndrome (9,10,32), FIT positivity rates at all thresholds were consistent with a large trial on FOB-gold in individuals with a similar advanced neoplasia prevalence (51), and baseline characteristics were detailed and not biased (the neoplasia and control group differed only in age and sex, which is unavoidable as these are known risk factors for neoplasia (11)). As our study population was under strict colonoscopy surveillance, the number of advanced neoplasia assessed was relatively small, leading to rather wide confidence intervals for sensitivity and PPV, although this effect was minimal for specificity and NPV. Nevertheless, our study may have been adequately powered to prove that FIT was noninferior to colonoscopy (15% absolute margin) in detecting CRC and advanced adenomas, according to the post hoc sample size calculation that was performed as part of the manuscript preparation process (see Supplementary Digital Content 1, http://links.lww.com/AJG/D384). The small number of advanced neoplasia also hampered comparison of median fecal hemoglobin concentration between advanced neoplasia types and resulted in only 2 stage I CRCs being included (the other 2 CRCs were stage III). A recent meta-analysis showed that stage I CRC, in particular T1 CRC, has a lower FIT sensitivity than later stages (52), although this meta-analysis did not evaluate very low thresholds (<10 μg Hb/g feces) and stage-specific sensitivity by tumor location. As detection of early-stage CRCs (stages I and II) is most relevant for reducing CRC morbidity and mortality, further larger studies should ensure that FIT at thresholds below 10 μg Hb/g feces can accurately detect both proximal and distal early-stage CRC in Lynch syndrome. Finally, because the Dutch guideline for Lynch syndrome does not recommend aspirin for chemoprevention, the impact of this agent on FIT performance remains to be determined.

In conclusion, the noninvasive FIT at positivity thresholds ≤4.1 μg Hb/g feces may be a promising strategy to postpone colonoscopy in approximately 9 of 10 individuals with Lynch syndrome. Large validation studies that also evaluate risk group-specific performance, patient acceptability and cost-effectiveness should be prioritized—in an effort to personalize colonoscopy surveillance in Lynch syndrome and thereby reduce colonoscopy burden as well as postcolonoscopy CRC rates.

## CONFLICTS OF INTEREST

**Guarantor of the article:** Dewkoemar Ramsoekh, MD, PhD.

**Specific author contributions:** E.L.S.A.v.L., N.K.H.d.B., M.E.v.L., E.D., M.A.J.M.J., J.J.K., J.P.K., M.C.W.S., B.C., and D.R.: conceived the study design. M.E.v.L., E.D., M.A.J.M.J., J.J.K., J.P.K., M.C.W.S., and D.R.: invited individuals to participate in the study. M.L.: analyzed the fecal immunochemical tests. E.L.S.A.v.L: collected the data, analyzed the data and drafted the manuscript. All authors critically revised the manuscript for important intellectual content. All authors approved the final version of the manuscript, including the authorship list.

**Financial support:** This study was funded by Dutch Digestive Foundation (MLDS); grant number WO 19-05. The funder of the study had no role in study design, data collection, data analysis, data interpretation, or writing of the report.

**Potential competing interests:** E.L.S.A.v.L., M.E.v.L., M.A.J.M.J., J.J.K., J.P.K., and M.L. declare no competing interests. N.K.H.d.B. has served as a speaker for AbbVie and MSD and has served as a consultant and principal investigator for TEVA Pharma BV and Takeda. He has received a research grant (unrestricted) from Dr. Falk, TEVA Pharma BV, Dutch Digestive Foundation (MLDS) and Takeda. E.D. has endoscopic equipment on a loan of FujiFilm and has received a research grant from FujiFilm. She has received an honorarium for a consultancy from FujiFilm, Olympus, InterVenn and Ambu, and speakers' fees from Olympus, GI Supply, Norgine, IPSEN, PAION and FujiFilm. G.A.M. is cofounder and board member (CSO) of CRCbioscreen BV. He has a research collaboration with CZ Health Insurances (cash matching to ZonMW grant) and he has research collaborations with Exact Sciences, Sysmex, Sentinel Ch. SpA, Personal Genome Diagnostics (PGDX), DELFi and Hartwig Medical Foundation; these companies provide materials, equipment, and/or sample/genomic analyses. G.A.M. is an Advisory Board member of “Missie Tumor Onbekend.” M.C.W.S. has received research support from Sysmex, Sentinel, Medtronic and Norgine. B.C. has several patents pending and/or issued. D.R. has received a research grant (unrestricted) from AbbVie. He has served as a member of the data safety monitoring board of the VIVIAD trial.

**Data availability statement:** The data that support the findings of this study are available from the corresponding author upon reasonable request.

**Study Highlights**

**WHAT IS KNOWN**

✓ The fecal immunochemical test (FIT) at thresholds ≤4 μg Hb/g feces has high sensitivity for sporadic colorectal cancer (CRC) and advanced adenomas.
✓ The role of FIT for Lynch syndrome has not yet been determined.

**WHAT IS NEW HERE**

✓ FIT at threshold 4.1 μg/g showed 89% sensitivity and 99% negative predictive value for CRC and advanced adenomas in Lynch syndrome.
✓ Per 100 FITs, 11 individuals would proceed to colonoscopy while 1 individual with advanced adenoma would be missed.
✓ FIT might enable personalized surveillance in Lynch syndrome, aiming to reduce colonoscopy overuse and postcolonoscopy CRC rates.

## Supplementary Material

## References

[1] Win AK Jenkins MA Dowty JG . Prevalence and penetrance of major genes and polygenes for colorectal cancer. Cancer Epidemiol Biomarkers Prev 2017;26(3):404–12. 27799157 10.1158/1055-9965.EPI-16-0693 PMC5336409

[2] van Leerdam ME Roos VH van Hooft JE . Endoscopic management of Lynch syndrome and of familial risk of colorectal cancer: European Society of Gastrointestinal Endoscopy (ESGE) guideline. Endoscopy 2019;51(11):1082–93. 31597170 10.1055/a-1016-4977

[3] Vasen H Hes F de Jong M. Dutch guideline for diagnostics and prevention of hereditary and familial tumours. 2017. (https://www.stoet.nl/wp-content/uploads/2017/04/STOET-Richtlijnenboekje-april2017_DEF.pdf). Accessed September 1, 2023.

[4] Denters MJ Schreuder M Depla AC . Patients' perception of colonoscopy: Patients with inflammatory bowel disease and irritable bowel syndrome experience the largest burden. Eur J Gastroenterol Hepatol 2013;25(8):964–72. 23660935 10.1097/MEG.0b013e328361dcd3

[5] van Liere E Jacobs IL Dekker E . Colonoscopy surveillance in Lynch syndrome is burdensome and frequently delayed. Fam Cancer 2023;22(4):403–11. 37171677 10.1007/s10689-023-00333-4 PMC10176312

[6] Engel C Vasen HF Seppala T . No difference in colorectal cancer incidence or stage at detection by colonoscopy among 3 countries with different Lynch syndrome surveillance policies. Gastroenterology 2018;155(5):1400–9.e2. 30063918 10.1053/j.gastro.2018.07.030

[7] Newton K Green K Lalloo F . Colonoscopy screening compliance and outcomes in patients with Lynch syndrome. Colorectal Dis 2015;17(1):38–46. 25213040 10.1111/codi.12778

[8] Peterse EFP Naber SK Daly C . Cost-effectiveness of active identification and subsequent colonoscopy surveillance of Lynch syndrome cases. Clin Gastroenterol Hepatol 2020;18(12):2760–7.e12. 31629885 10.1016/j.cgh.2019.10.021 PMC7162709

[9] Houwen B Hazewinkel Y Pellise M . Linked colour imaging for the detection of polyps in patients with Lynch syndrome: A multicentre, parallel randomised controlled trial. Gut 2022;71(3):553–60. 34086597 10.1136/gutjnl-2020-323132 PMC8862075

[10] Rivero-Sanchez L Arnau-Collell C Herrero J . White-light endoscopy is adequate for Lynch syndrome surveillance in a randomized and noninferiority study. Gastroenterology 2020;158(4):895–904.e1. 31520613 10.1053/j.gastro.2019.09.003

[11] Dominguez-Valentin M Sampson JR Seppala TT . Cancer risks by gene, age, and gender in 6350 carriers of pathogenic mismatch repair variants: Findings from the Prospective Lynch Syndrome Database. Genet Med 2020;22(1):15–25. 31337882 10.1038/s41436-019-0596-9 PMC7371626

[12] de Jonge L Worthington J van Wifferen F . Impact of the COVID-19 pandemic on faecal immunochemical test-based colorectal cancer screening programmes in Australia, Canada, and the Netherlands: A comparative modelling study. Lancet Gastroenterol Hepatol 2021;6(4):304–14. 33548185 10.1016/S2468-1253(21)00003-0 PMC9767453

[13] Senore C Basu P Anttila A . Performance of colorectal cancer screening in the European Union Member States: Data from the second European screening report. Gut 2019;68(7):1232–44. 30530530 10.1136/gutjnl-2018-317293

[14] Digby J Cleary S Gray L . Faecal haemoglobin can define risk of colorectal neoplasia at surveillance colonoscopy in patients at increased risk of colorectal cancer. United European Gastroenterol J 2020;8(5):559–66. 10.1177/2050640620913674 PMC7268942 32213041

[15] Katsoula A Paschos P Haidich AB . Diagnostic accuracy of fecal immunochemical test in patients at increased risk for colorectal cancer: A meta-analysis. JAMA Intern Med 2017;177(8):1110–8. 28628706 10.1001/jamainternmed.2017.2309 PMC5710432

[16] Berwald G Young GP Cock C . The diagnostic performance of fecal immunochemical tests for detecting advanced neoplasia at surveillance colonoscopy. Clin Gastroenterol Hepatol 2024;22(4):878–85.e2. 37743036 10.1016/j.cgh.2023.09.016

[17] de Klaver W Wisse PHA van Wifferen F . Clinical validation of a multitarget fecal immunochemical test for colorectal cancer screening: A diagnostic test accuracy study. Ann Intern Med 2021;174(9):1224–31. 34280333 10.7326/M20-8270

[18] Kaminski MF Thomas-Gibson S Bugajski M . Performance measures for lower gastrointestinal endoscopy: A European Society of Gastrointestinal Endoscopy (ESGE) Quality Improvement Initiative. Endoscopy 2017;49(4):378–97. 28268235 10.1055/s-0043-103411

[19] Rivero-Sanchez L Gavric A Herrero J . The “diagnose and leave in” strategy for diminutive rectosigmoid polyps in Lynch syndrome: A post hoc analysis from a randomized controlled trial. Endoscopy 2022;54(1):27–34. 33271604 10.1055/a-1328-5405

[20] Dixon MF. Gastrointestinal epithelial neoplasia: Vienna revisited. Gut 2002;51(1):130–1. 12077106 10.1136/gut.51.1.130 PMC1773259

[21] Nagtegaal ID Odze RD Klimstra D . The 2019 WHO classification of tumours of the digestive system. Histopathology 2020;76(2):182–8. 31433515 10.1111/his.13975 PMC7003895

[22] Gupta S Lieberman D Anderson JC . Recommendations for follow-up after colonoscopy and polypectomy: A consensus update by the US Multi-Society Task Force on Colorectal Cancer. Gastroenterology 2020;158(4):1131–53.e5. 32044092 10.1053/j.gastro.2019.10.026 PMC7672705

[23] Hassan C Antonelli G Dumonceau JM . Post-polypectomy colonoscopy surveillance: European Society of Gastrointestinal Endoscopy (ESGE) guideline: Update 2020. Endoscopy 2020;52(8):687–700. 32572858 10.1055/a-1185-3109

[24] van Toledo D IJspeert J Spaander M . Colorectal cancer risk after removal of polyps in fecal immunochemical test based screening. eClinicalMedicine 2023;61:102066. 37528844 10.1016/j.eclinm.2023.102066 PMC10388570

[25] Fagan TJ. Letter: Nomogram for Bayes's theorem. N Engl J Med 1975;293(5):257. 1143310 10.1056/NEJM197507312930513

[26] Selby K Levine EH Doan C . Effect of sex, age, and positivity threshold on fecal immunochemical test accuracy: A systematic review and meta-analysis. Gastroenterology 2019;157(6):1494–505. 31472152 10.1053/j.gastro.2019.08.023 PMC6878177

[27] Lincoln AG Benton SC Piggott C . Risk-stratified faecal immunochemical testing (FIT) for urgent colonoscopy in Lynch syndrome during the COVID-19 pandemic. BJS Open 2023;7(5):zrad079. 37668669 10.1093/bjsopen/zrad079 PMC10478750

[28] van Toledo D Breekveldt ECH IJspeert JEG . Advanced serrated polyps as a target of screening: Detection rate and positive predictive value within a fecal immunochemical test-based colorectal cancer screening population. Endoscopy 2023;55(6):526–34. 36323332 10.1055/a-1971-3488

[29] Chang LC Shun CT Hsu WF . Fecal immunochemical test detects sessile serrated adenomas and polyps with a low level of sensitivity. Clin Gastroenterol Hepatol 2017;15(6):872–9.e1. 27498176 10.1016/j.cgh.2016.07.029

[30] Cock C Anwar S Byrne SE . Low sensitivity of fecal immunochemical tests and blood-based markers of DNA hypermethylation for detection of sessile serrated adenomas/polyps. Dig Dis Sci 2019;64(9):2555–62. 30835026 10.1007/s10620-019-05569-8

[31] Edelstein DL Axilbund J Baxter M . Rapid development of colorectal neoplasia in patients with Lynch syndrome. Clin Gastroenterol Hepatol 2011;9(4):340–3. 21070872 10.1016/j.cgh.2010.10.033 PMC3073674

[32] Vleugels JLA Sahin H Hazewinkel Y . Endoscopic detection rate of sessile serrated lesions in Lynch syndrome patients is comparable with an age- and gender-matched control population: Case-control study with expert pathology review. Gastrointest Endosc 2018;87(5):1289–96. 29233671 10.1016/j.gie.2017.11.034

[33] Meester RGS van Herk M Lansdorp-Vogelaar I . Prevalence and clinical features of sessile serrated polyps: A systematic review. Gastroenterology 2020;159(1):105–18.e25. 32199884 10.1053/j.gastro.2020.03.025 PMC8653879

[34] Rex DK Ahnen DJ Baron JA . Serrated lesions of the colorectum: Review and recommendations from an expert panel. Am J Gastroenterol 2012;107(9):1315–29; quiz 1314, 1330. 22710576 10.1038/ajg.2012.161 PMC3629844

[35] Holme O Bretthauer M Eide TJ . Long-term risk of colorectal cancer in individuals with serrated polyps. Gut 2015;64(6):929–36. 25399542 10.1136/gutjnl-2014-307793

[36] Redwood DG Asay ED Blake ID . Stool DNA testing for screening detection of colorectal neoplasia in Alaska native people. Mayo Clin Proc 2016;91(1):61–70. 26520415 10.1016/j.mayocp.2015.10.008

[37] Kapidzic A van Roon AH van Leerdam ME . Attendance and diagnostic yield of repeated two-sample faecal immunochemical test screening for colorectal cancer. Gut 2017;66(1):118–23. 26370109 10.1136/gutjnl-2014-308957

[38] Wieten E de Klerk CM Lansdorp-Vogelaar I . A quarter of participants with advanced neoplasia have discordant results from 2-sample fecal immunochemical tests for colorectal cancer screening. Clin Gastroenterol Hepatol 2020;18(8):1805–11.e1. 31563557 10.1016/j.cgh.2019.09.024

[39] Imperiale TF Ransohoff DF Itzkowitz SH . Multitarget stool DNA testing for colorectal-cancer screening. N Engl J Med 2014;370(14):1287–97. 24645800 10.1056/NEJMoa1311194

[40] Lepore Signorile M Disciglio V Di Carlo G . From genetics to histomolecular characterization: An insight into colorectal carcinogenesis in Lynch syndrome. Int J Mol Sci 2021;22(13):6767. 34201893 10.3390/ijms22136767 PMC8268977

[41] Kastrinos F Ingram MA Silver ER . Gene-specific variation in colorectal cancer surveillance strategies for Lynch syndrome. Gastroenterology 2021;161(2):453–62.e15. 33839100 10.1053/j.gastro.2021.04.010 PMC9330543

[42] Suerink M Rodriguez-Girondo M van der Klift HM . An alternative approach to establishing unbiased colorectal cancer risk estimation in Lynch syndrome. Genet Med 2019;21(12):2706–12. 31204389 10.1038/s41436-019-0577-z

[43] Ten Broeke SW van der Klift HM Tops CMJ . Cancer risks for PMS2-associated Lynch syndrome. J Clin Oncol 2018;36(29):2961–8. 30161022 10.1200/JCO.2018.78.4777 PMC6349460

[44] Winkels RM Botma A Van Duijnhoven FJ . Smoking increases the risk for colorectal adenomas in patients with Lynch syndrome. Gastroenterology 2012;142(2):241–7. 22062356 10.1053/j.gastro.2011.10.033

[45] Movahedi M Bishop DT Macrae F . Obesity, aspirin, and risk of colorectal cancer in carriers of hereditary colorectal cancer: A prospective investigation in the CAPP2 study. J Clin Oncol 2015;33(31):3591–7. 26282643 10.1200/JCO.2014.58.9952

[46] Robertson DJ Lee JK Boland CR . Recommendations on fecal immunochemical testing to screen for colorectal neoplasia: A consensus statement by the US Multi-Society Task Force on Colorectal Cancer. Am J Gastroenterol 2017;112(1):37–53. 27753435 10.1038/ajg.2016.492

[47] Eiken Chemical. Faecal immunochemical test. (https://www.eiken.co.jp/en/products/fit/ceres/). Accessed July 1, 2024.

[48] Gonzalez-Lopez N Quintero E Gimeno-Garcia AZ . Screening uptake of colonoscopy versus fecal immunochemical testing in first-degree relatives of patients with non-syndromic colorectal cancer: A multicenter, open-label, parallel-group, randomized trial (ParCoFit study). PLoS Med 2023;20(10):e1004298. 37874831 10.1371/journal.pmed.1004298 PMC10597530

[49] van Liere E de Boer NKH Parsan EA . Effect of the COVID-19 pandemic on endoscopic surveillance in Lynch syndrome in the Netherlands. Lancet Gastroenterol Hepatol 2023;8(6):504–6. 10.1016/S2468-1253(23)00067-5 PMC10075316 37030312

[50] Sanchez A Roos VH Navarro M . Quality of colonoscopy is associated with adenoma detection and postcolonoscopy colorectal cancer prevention in Lynch syndrome. Clin Gastroenterol Hepatol 2022;20(3):611–21.e9. 33157315 10.1016/j.cgh.2020.11.002

[51] Grobbee EJ van der Vlugt M van Vuuren AJ . A randomised comparison of two faecal immunochemical tests in population-based colorectal cancer screening. Gut 2017;66(11):1975–82. 27507905 10.1136/gutjnl-2016-311819

[52] Niedermaier T Balavarca Y Brenner H. Stage-specific sensitivity of fecal immunochemical tests for detecting colorectal cancer: Systematic review and meta-analysis. Am J Gastroenterol 2020;115(1):56–69. 31850933 10.14309/ajg.0000000000000465 PMC6946106

