# Supplementary material for: Fecal Immunochemical Test to Detect Colorectal Neoplasia in Lynch Syndrome: A Prospective Multicenter Study
Source: Am J Gastroenterol. 2024 Aug 20;120(3):632–41. doi: 10.14309/ajg.0000000000003043 (PMC11864054; doi:10.14309/ajg.0000000000003043)
Supplement: Supplementary file 1 [file acg-120-632-s001.docx]

**TABLE OF CONTENTS**

| **Topic** | **Page** |
| --- | --- |
| Table S1. Characteristics of relevant precancerous neoplasia at colonoscopy | 2 |
| Figure S1. Fagan’s nomogram of all relevant neoplasia | 3 |
| Figure S2. Fagan’s nomogram of advanced neoplasia | 4 |
| Power calculation | 5 |

**Table S1.** Characteristics of relevant precancerous neoplasia at colonoscopy

|  | **Advanced adenomas**  (n=5) | **Non-advanced adenomas** (n=57) | **Advanced serrated lesions** (n=4) |
| --- | --- | --- | --- |
| **Location**, *n*  Caecum  Ascending, incl. hepatic flexure  Transverse  Descending, incl. splenic flexure  Sigmoid  Rectum | -  -  1  1  2  1 | 4  16  13  11  11  2 | -  1  1  1  -  1 |
| **Size**, *median (interquartile range)* | 15 mm (11 – 15)^1^ | 3 mm (2 – 4) | 11 mm (5 – 14)^1^ |
| **Paris classification for morphology**^2^, *n* Pedunculated (Ip)  Sub-pedunculated (Isp)  Sessile (Is)  Flat or flat elevated (IIa or IIb)  Depressed (IIc) | 2  -  1  2  - | 2  -  32  15  - | -  -  4  -  - |
| **High-grade dysplasia**, *n* | 0 | n.a. | n.a. |
| **Villous component**, *n* | 1 | n.a. | n.a. |
| **Type of serrated lesion**, *n*  Sessile serrated lesion without dysplasia  Sessile serrated lesion with dysplasia | n.a. | n.a. | 3  1 |

1: The size of the five advanced adenomas separately was: 10, 12, 15, 15 and 15 mm. The size of the four advanced serrated lesions was: 3, 10, 12 and 15 mm
2: Missing values for n=8 non-advanced adenomas.

**Figure S1.** Fagan’s nomogram illustrating the pre-test and post-test probability of all relevant neoplasia (colorectal cancer, advanced serrated lesions and all adenomas), following positive (blue line) and negative (red line) FIT at different thresholds. This figure shows that the estimated probability of having relevant neoplasia following negative FIT at thresholds ≤20 μg Hb/g feces was more or less comparable to the pre-test probability (grey line, representing the relevant neoplasia prevalence), meaning that a negative FIT did not change the probability that an individual had relevant neoplasia. *FIT = fecal immunochemical test; NLR = negative likelihood ratio; PLR = positive likelihood ratio.*

**
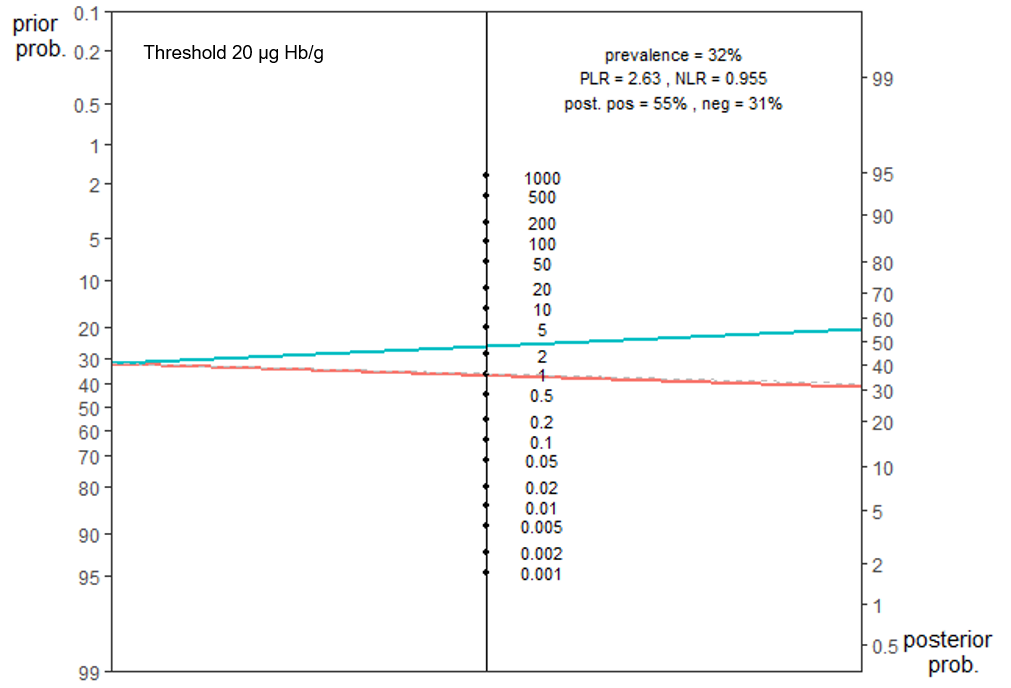

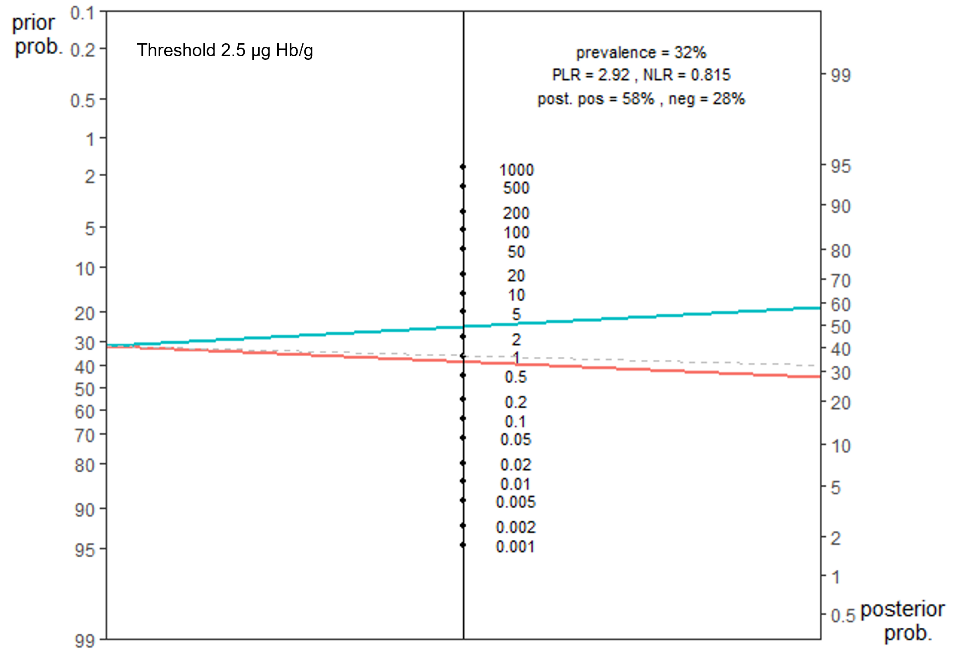

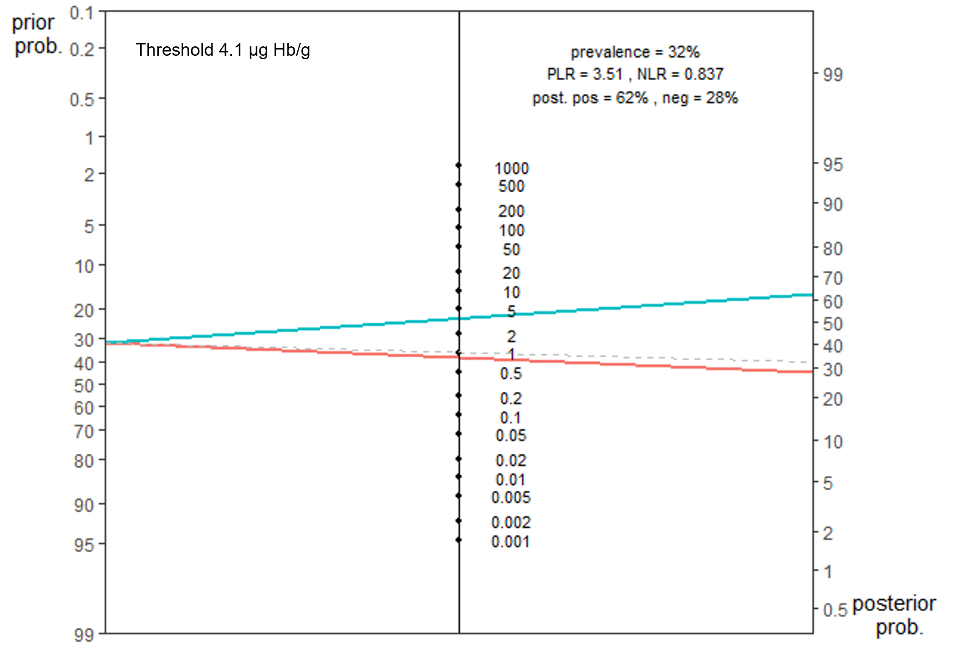
**


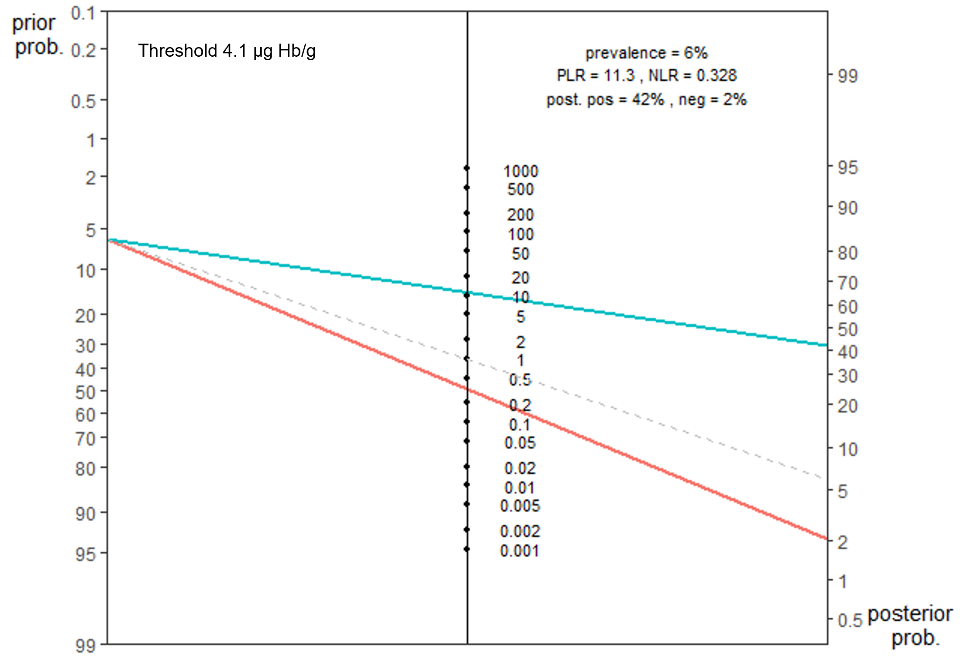

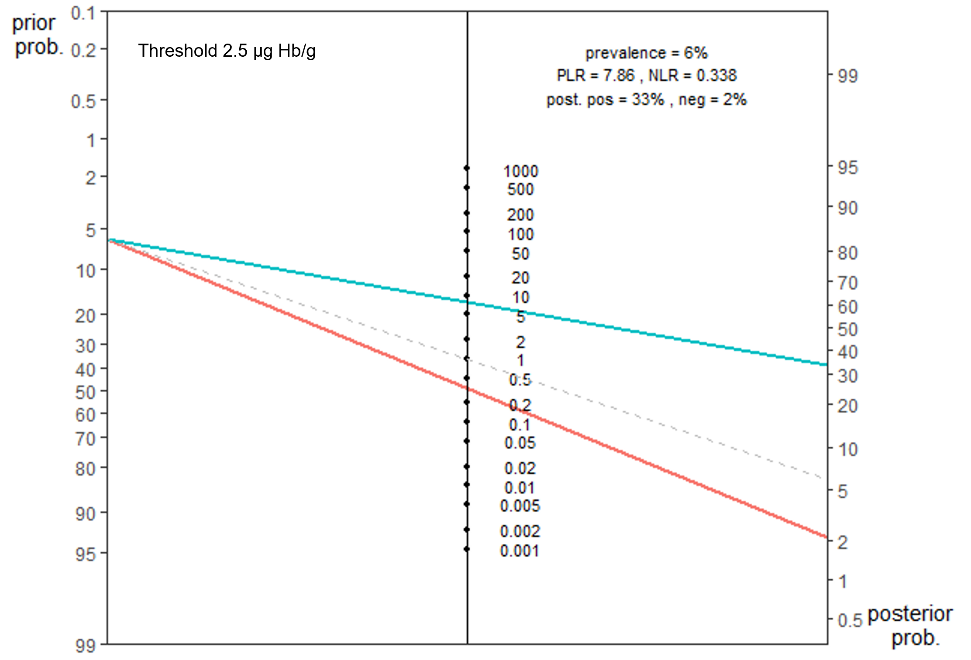
**Figure S2.** Fagan’s nomogram illustrating the pre-test and post-test probability of advanced neoplasia (colorectal cancer, advanced adenomas and advanced serrated lesions), following positive (blue line) and negative (red line) FIT at different thresholds. The prevalence of advanced neoplasia was considered as the pre-test probability, and the grey line represents the situation when the post-test probability remained unchanged. The figure shows that a negative FIT at either low thresholds decreased the estimated probability of advanced neoplasia from 6% to 2%, while a positive FIT increased the probability of these neoplasia to 33% (2.5 μg Hb/g feces threshold) or 42% (4.1 μg Hb/g feces threshold). In contrast, probability of advanced neoplasia after negative FIT with threshold 20 μg Hb/g feces was 4%. *FIT = fecal immunochemical test; NLR = negative likelihood ratio; PLR = positive likelihood ratio.*


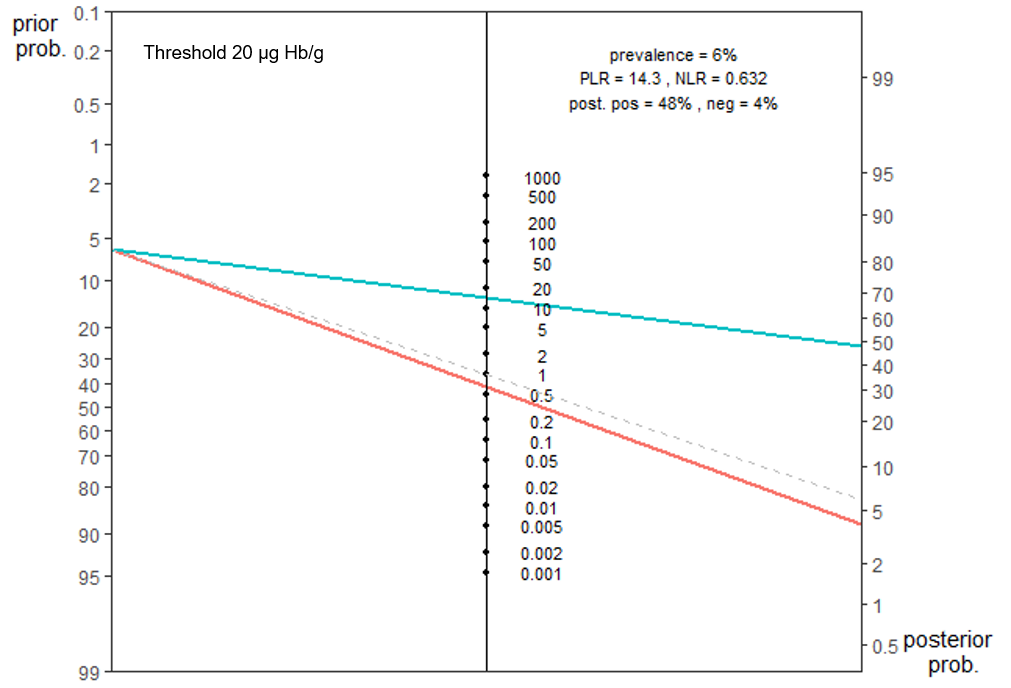


**Power calculation**

The current study was performed as part of a large longitudinal study on biomarkers for colorectal neoplasia detection in Lynch syndrome, and therefore did not include a sample size calculation prior to the start of the study.

To interpret the results of our study, we believe it is important to assess whether our study may have been adequately powered. Therefore, we conducted a sample size calculation as part of the manuscript preparation process, using a non-inferiority test for paired dichotomous data with 80% power and a one-sided significance level of 5%. The non-inferiority margin for the absolute difference in detection of colorectal cancer (CRC) and advanced adenomas (AA) between colonoscopy and fecal immunochemical test (FIT) was set at 15%. Based on previous research, we assumed that with FIT at the lower limit of quantitation the nuisance parameter would be 40%^1^. Under the alternative hypothesis of equal positive tests by the two methods (i.e. the actual absolute difference being 0%), 112 individuals would have been required to prove non-inferiority.

In our study of 217 individuals we found that FIT at the lower limit of quantitation had a 89% sensitivity and 91% specificity to detect CRC and AA. As a result, the absolute difference in detection of CRC and AA between FIT and colonoscopy was 7.7% with a 90% confidence interval ranging between 2.0 – 13.4%. These percentages did not cross the non-inferiority margin, hence, our study may indicate with sufficient power that FIT at the lower limit of quantitation may be non-inferior to colonoscopy in detecting CRC and AA in Lynch syndrome.

1. Digby J, Cleary S, Gray L, et al. Faecal haemoglobin can define risk of colorectal neoplasia at surveillance colonoscopy in patients at increased risk of colorectal cancer. *United European Gastroenterol J*. Jun 2020;8(5):559-566. doi:10.1177/2050640620913674
